# The Study of Magnetic Properties for Non-Magnetic Ions Doped BiFeO_3_

**DOI:** 10.3390/ma14154061

**Published:** 2021-07-21

**Authors:** Yongtao Li, Liqing Liu, Dehao Wang, Hongguang Zhang, Xuemin He, Qi Li

**Affiliations:** 1 School of Science, Nanjing University of Posts and Telecommunications, Nanjing 210023, China; liyt@njupt.edu.cn (Y.L.); Zhanghongguang2003@126.com (H.Z.); hxm@njupt.edu.cn (X.H.); 2 College of Electronic Science and Engineering, Nanjing University of Posts and Telecommunications, Nanjing 210023, China; 1218022814@njupt.edu.cn; 3 New Energy Technology Engineering Laboratory of Jiangsu Province, Nanjing 210023, China; 4 School of Physics, Southeast University, Nanjing 211189, China; qli@seu.edu.cn

**Keywords:** magnetic properties, XAFS, local structure, sol-gel method

## Abstract

BiFeO_3_ is considered as a single phase multiferroic. However, its magnetism is very weak. We study the magnetic properties of BiFeO_3_ by Cu and (Cu, Zn). Polycrystalline samples Bi(Fe_0.95_Cu_0.05_)O_3_ and BiFe_0.95_(Zn_0.025_Cu_0.025_)O_3_ are prepared by the sol-gel method. The magnetic properties of BiFe_0.95_(Zn_0.025_Cu_0.025_)O_3_ are greater than that of BiFeO_3_ and Bi(Fe_0.95_Cu_0.05_)O_3_. The analyses of X-ray absorption fine structure data show that the doped Cu atoms well occupy the sites of the Fe atoms. X-ray absorption near edge spectra data confirm that the valence state of Fe ions does not change. Cu and Zn metal ion co-doping has no impact on the local structure of the Fe and Bi atoms. The modification of magnetism by doping Zn can be understood by the view of the occupation site of non-magnetically active Zn^2+^.

## 1. Introduction

BiFeO_3_ (BFO) is considered a prototype multiferroic material and is probably the most studied multiferroic material as it is the most promising candidate for realizing multiferroic devices [1,2]. However, BFO has some inherent problems, such as weak magnetism, a high leakage current, lower magnetoelectric coupling coefficients.

The magnetic ordering of BFO at room temperature was proposed by Sosnowska [3], and later confirmed to be an antiferromagnetic ordering which is modulated with a period of 62 nm [4,5]. To solve the problem of weak magnetism, several attempts have been made by suitable modifications at the Bi and/or Fe sites substitution or fabrication of composites [6,7,8,9]. The weak magnetic characteristics of BFO are attributed to some factors, such as spiral spin structure, orientation of magnetic moments perpendicular to the rhombohedral axis and magnetic moment canting [10]. Substitution at Bi sites by rare earth ions releases the latent magnetization resulting in improvement of magnetic properties, which is attributed to structural phase transition [11,12,13]. Transition metal element cobalt has been used to dope into the Fe-site of BFO to enhance ferromagnetism, and the origin of the enhancement of the saturated magnetization is related to the occupation of the Bi site of crystal lattice [14,15,16].

At present, the study of magnetic properties for non-magnetic ions doped-BFO is scarce. Wei et al. [17] argued that the replacement of Fe^3+^ by Zr^4+^ locally breaks the antiferromagnetic superexchange, allowing a macroscopic magnetization contribution in BiFe_0.9_Zr_0.1_O_3_. The co-doping of BFO with nomagnetic Y and Zr ions reduces leakage current and induces ferromagnetic properties [18]. The introduction of non-magnetic Sn into BFO thin film results in the weakness of magnetism [19]. Zn ion, as a 3*d* transitional metal ion with no spin moment, has been studied in the magnetic property of Zn-doped BFO. For BiFe_0.95_Zn_0.05_O_3_ sample, due to the intervention of Zn atoms in the Fe atom chains, the antiferromagnetic spin chains of Fe ions will be broken, and the paramagnetic properties will be enhanced [20]. BiFe_1__-_*_x_*Zn*_x_*O_3_ (*x* = 0.1–0.2) samples exhibit a weak ferrimagnetic nature at 300 K and superparamagnetic nature at 5 K [21]. First-principles investigation on magnetic properties of Cu and Zn doped BFO that non-magnetic Cu and Zn doping leads to the diversity and complexity of magnetic properties [22].

In this paper, we will use the X-ray Absorption Fine Structure (XAFS) technique to research the local structure and magnetic properties of the samples in which the non-magnetically active Zn^2+^ ions and Cu^2+^ ions are doped into Fe-sites of BFO.

## 2. Experiments

Polycrystalline samples Bi(Fe_0.95_Cu_0.05_)O_3_ (BFC) and BiFe_0.95_(Zn_0.025_Cu_0.025_)O_3_ (BFZC) with nominal doping ratio were prepared by the sol-gel method using the raw materials of Bi_2_O_3_, Zn(CHOOCH)_2_, Cu(CHOOCH)_2_, and Fe(NO_3_)_3_ taken in desired cation ratios. Tartaric acid (the amount of tartaric acid is equal to the total metallic ions in the precursor solutions) was added as a chelating agent. To obtain gel state, the solution was mixed thoroughly using the magnetic stirrer and baked at 80 °C for 24 h. The green bodies were then pre-sintered at 250 °C. After that, the pre-sintered powder was annealed at 600 °C for 2 h in air. Finally, the samples were slowly cooled down to room temperature.

The crystal structures of the samples were examined by X-ray diffraction (XRD) (Rigaku Smartlab, Japan) patterns with Cu-*K*_α_ radiation (*λ* = 1.5406 Å). Magnetic measurements were performed with a physical property measurement system (PPMS-9, Quantum Design, USA). Fe-*K*, Cu-*K,* and Bi-*L*_3_ edge XAFS data were collected at the beamline (1W1B) of Beijing Synchrotron Radiation Facility—(BSRF), Beijing, China.

## 3. Results and Discussions

**Figure 1:**
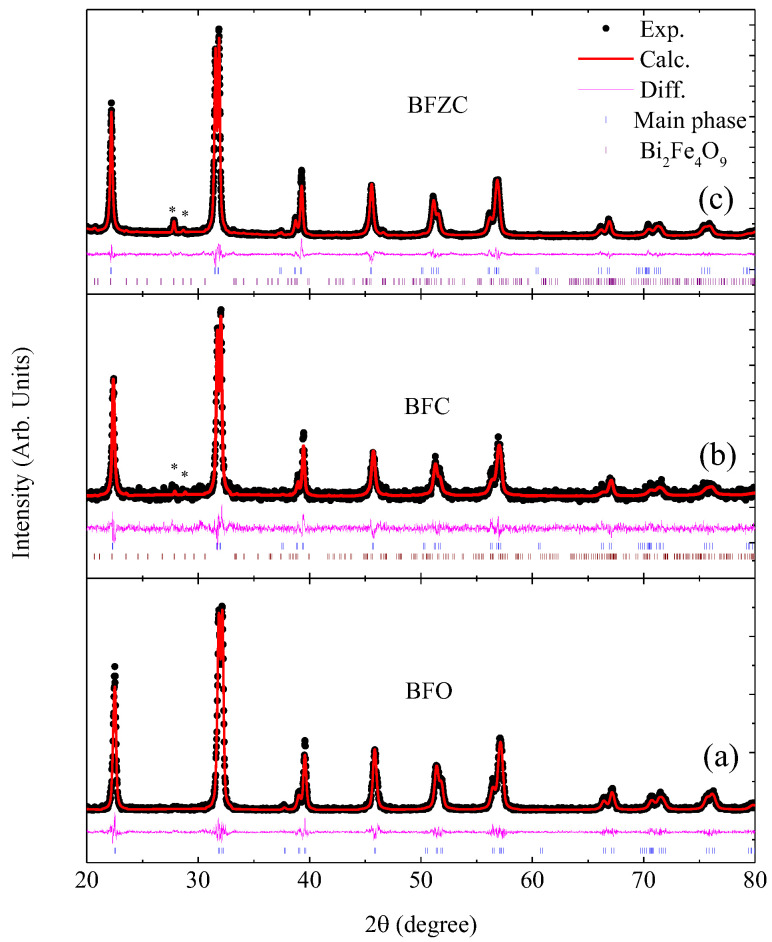
The refined XRD patterns of the samples. * indicate some peaks, characteristic of Bi_2_Fe_4_O_9_ phases. (**a**) BFO, (**b**) BFC, (**c**) BFZC.

Figure 1 shows the XRD patterns of all the samples. It can be seen that all the samples are mainly in single phase. According to the positions of the Bragg reflections, all these samples belong to a rhombohedral structure with group R3c. A small amount of secondary phases was observed in BFC and BFZC. According to the Rietveld refinement, the secondary phases were indexed into Bi_2_Fe_4_O_9_ [23]. The percentage of it is 0.96% and 3.94% for BFC and BFZC, respectively. The lattice constants are listed in Table 1.

**Figure 2:**
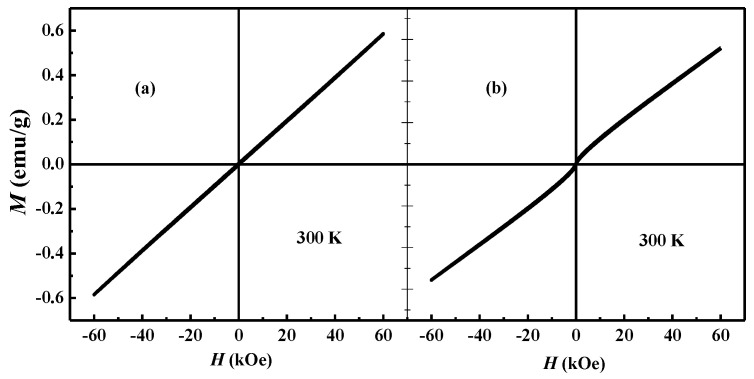
The magnetic hysteresis loop of the samples at 300 K. (**a**) BFC, (**b**) BFZC.

A comparison between the magnetic hysteresis loop of BFC and of BFZC measured at 300 K is shown in Figure 2. BFC exhibits a near linear *M*-*H* relationship, which is similar to that of BFO, as shown in Figure 2a, based on the unsaturated magnetization curves in the fields up to 60 kOe. In Figure 2b, the magnetic hysteresis curve of the sample BFZC is nearly similar to that of BFC, but the former shows a tiny “S”-shape in the range of low magnetic field. It indicates the sample BFZC presents antiferromagnetism, along with very weak ferromagnetic characteristics.

**Figure 3:**
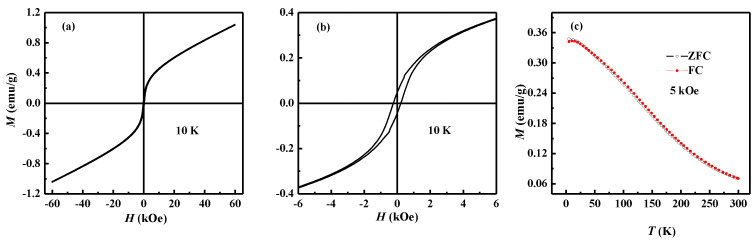
(**a**) The magnetic hysteresis loop of BFZC at 10 K. (**b**) Enlarged *M*-*H* curve at 10 K. (**c**) ZFC curves under a field of 5 kOe and FC curve of the sample cooled under a magnetic field of 5 kOe.

The magnetic hysteresis loop of BFZC at 10 K is plotted in Figure 3a. The magnetic property of the sample is evidently improved compared with that of BFO. The ferromagnetic characteristics at low temperatures are more obvious than those at room temperature. A well-developed *M*-*H* loop together with a small but nonzero remnant magnetization can be observed at low temperature, as shown in Figure 3b.

To understand the origin of the observed ferromagnetism in the BFZC sample at low temperatures, zero field cooled (ZFC) and field cooled (FC) temperature dependent magnetization curves were measured under 5 kOe from 10–300 K, as shown in Figure 3c. There is no divergence between FC and ZFC magnetization curves, which indicates conventional spin glass behavior does not exist in the BFZC sample.

**Figure 4:**
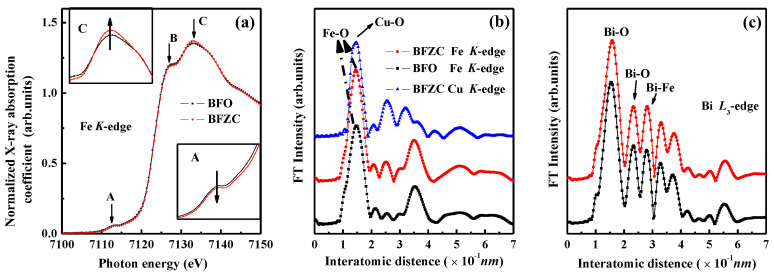
The XAFS data of the BFO and BFZC samples. (**a**) Fe-*K* XANES spectra. (**b**) Fourier transforms of BFO and BFZC obtained by Fe-*K* and Cu-*K* XAFS. (**c**) Fourier transforms of BFO and BFZC obtained by Bi-*L*_3_ XAFS.

The X-ray absorption near edge structure (XANES) spectra of Fe *K*-edge for the BFO and BFZC samples are plotted in Figure 4a. It can be seen that the observed spectra of both compounds are very similar. Each spectrum includes a pre-edge peak A, a shoulder peak B, and main peak C, which demonstrates that Zn^2+^, Cu^2+^ co-doping has a weak influence on the microscopic local structure around the Fe atoms. The absorption energy position does not shift, which suggests that the valence state of Fe ions does not change after Zn and Cu ions co-doping. The pre-edge feature A of Fe *K*-edge denotes the electronic excitation of Fe from the core state (1*s*) to an unoccupied orbital (3*d*), whereas peak C corresponds to the 1*s*-4*d* dipole-allowed transition. As a shoulder peak, peak B is caused by ligand-to-metal charge transfer process in which the oxygen 2*p* electron partially transfers to the Fe 3*d* orbital. It is worth noting that the peak intensity of the peaks A weakly increases and that of the peak C slightly decreases due to (Zn, Cu)-co-doping, as shown in the insets of Figure 4a. This unobvious converting of the relative peak intensity of peak A and C indicates that a weak structure distortion induces the 3*d*-4*p* orbital hybridization [24]. This weak distortion of structure is insufficient to cause local atomic structure greatly changing.

Figure 4b exhibits the Fourier transformed curves of BFO and BFZC obtained by Fe-*K* and Cu-*K* edge XAFS spectra. However, compared to the actual interatomic distance, the peak position will shift approximately 0.05 nm shorter because no phase-shift correction is considered for Fourier transformation [25]. The main peaks located at 0.147 nm are assigned to the Fe-O bond for BFO a BFZC, which is the first neighbor coordination shell peak. It can give the information of FeO_6_ octahedron. Compared to the main peak of BFO, when Zn and Cu ions are doped, the position and intensity of Fe-O peak for BFZC hardly change. It indicates that the doping by both Zn and Cu ions does not affect the local structure of central Fe atom in this system [26]. This result is in good agreement with that of Fe *K*-edge XANES spectra. It can be seen that the shape of radial distribution function curve of Cu *K*-edge is almost identical to that of Fe *K*-edge, which illustrates that the Cu ions occupy Fe-sites in this sample. Comparing the Fourier transform of Fe and Cu *K*-edge XAFS data of BFZC sample, it is noteworthy that the intensity and shape of the first coordination shell peak do not change significantly, but the second coordination shell peak presents a clear change. It exhibits that Cu ion substitution mainly affects the second coordination shell.

The Bi *L*_3_-edge EXAFS spectra of the BFZC sample show an analogous pattern to that of BFO sample, as shown in Figure 4c, which substantiates the fact that doping by Cu and Zn ions has no or very little impact on the local structure of Bi atoms.

From the analysis of the magnetic properties of the samples, we can conclude that the magnetic properties of Cu-doped BFO is not enhanced, similar to that of BFO, although Cu^2+^ ions possess spin moment. When non-magnetic transition metal Zn ion is doped into BFC, a larger magnetic character of the sample is exhibited.

**Figure 5:**
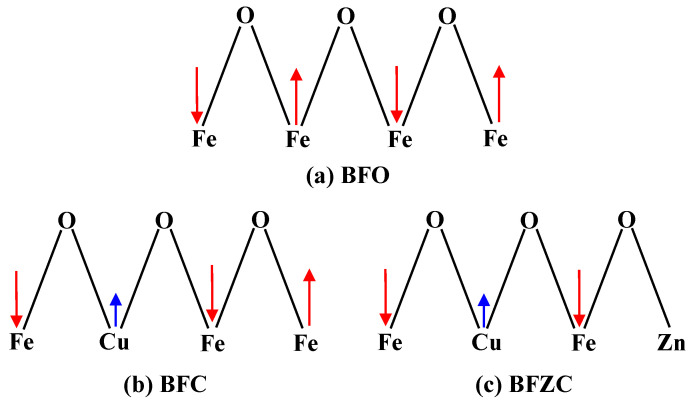
Scheme of the spins arrangement on pure (**a**) BFO, (**b**) BFC, and (**c**) BFZC.

It is known that the magnetic property of BFO is associated with the spins of ions at Fe-sites. The magnetic ordering of this series of samples based on parent BFO is essentially G-type antiferromagnetic with cycloidal spin magnetic ordering [3,8]. The magnetic moments of the Fe cations produce antiparallel alignment through Fe-O-Fe superexchange interactions, as shown in Figure 5a. The analysis of EXAFS data has shown that the Cu atoms well occupy the sites of the Fe atoms. However, Cu^2+^ has a weaker magnetic moment compared to Fe^3+^ ion due to the electronic configuration of the Cu^2+^. The number of the incorporated Cu^2+^ is limited, thus the magnetic property of the BFC sample is not strong, as shown in Figure 5b. Further doping by non-magnetic transition metal ions (Zn^2+^) at Fe sites gives rise to a loss of one of the spin moments in the magnetic Fe ion spin chain, leading to a transformation of the magnetic moment around substitution sites in the whole spin chain from originally antiparallel to parallel or nearly parallel, as shown in Figure 5c, i.e., a ferromagnetic character forms. Consequently, the magnetic property of BFZC sample is enhanced when the non-magnetically active Zn^2+^ ions are further doped.

In addition to the effect of non-magnetic Zn ions and weakly magnetic Cu ions on the spin arrangement of antiferromagnetic Fe, other factors may also affect the magnetic properties. (1) Particle size. The average particle size of polycrystalline BFO prepared by the sol-gel method is usually in dozens of nanometers, which is lower than the periodicity of a spin cycloid (62 nm) [27]. The destruction of the periodicity of the spin cycloid enhances the magnetization [28]. (2) The presence of magnetic impurity phase. From XRD, there is an amount of Bi_2_Fe_4_O_9_ phase detected, especially in BFZC samples, which will improve the macro-magnetism [28]. (3) Crystal structural transition and local structural distortion. As reported, the spiral spin modulation will be destroyed by the structural transition and be suppressed by the larger distortion of the lattice [29]. From the analysis of XRD and XAFS, there is no change of crystal structure and local lattice distortion by doping. Therefore, this factor can be excluded.

## 4. Conclusions

In summary, BFO, BFC, and BFZC samples have been prepared by the sol-gel method. XRD patterns demonstrate that all samples present well single phase. The magnetic property of BFZC is stronger than that of BFO. XANES data analysis confirms that the valence states of iron ions do not change. The EXAFS data analysis shows that the Cu ions have been completely incorporated into the BiFeO_3_ structure and have occupied Fe-sites, and that non-magnetic transition metal ion doping has no impact on the local structure of the Fe and Bi atoms. Finally, the magnetic property improvement of (Cu, Zn)-co-doped sample is explained from the perspective of the occupation by non-magnetic transition metal ions.

## Figures and Tables

**Table 1:** The lattice constants a, c, and relevant bond lengths R(Bi-O) from XRD and r(Bi-O) from XAFS.

	BFO	BFC	BFZC
a(Å)	5.58092 ± 0.00018	5.58143 ± 0.00026	5.58602 ± 0.0007
c(Å)	13.87368 ± 0.00084	13.86918 ± 0.00128	13.88424 ± 0.00035
R(Bi-O1) (Å)	1.92675 ± 0.00006	1.95260 ± 0.00008	2.01782 ± 0.00002
r(Bi-O) (Å)	1.94194 ± 0.02	1.95234 ± 0.02	1.97262 ± 0.02

## Data Availability

The data can be provided by authors on request.
